# Utilizing the Lactate Dehydrogenase-to-Albumin Ratio for Survival Prediction in Patients with Neuroblastoma

**DOI:** 10.3390/children13020220

**Published:** 2026-02-04

**Authors:** Suwen Li, Yue Ma, Shan Wang

**Affiliations:** 1 Department of Oncological Surgery, Children’s Hospital of Chongqing Medical University, National Clinical Research Center for Children and Adolescents’ Health and Diseases, Ministry of Education Key Laboratory of Child Development and Disorders, Chongqing 400014, China; leesoon0422@hotmail.com (S.L.); mymy9527@yeah.net (Y.M.); 2 International Science and Technology Cooperation Base of Child Development and Critical Disorders, Chongqing 400014, China; 3 Chongqing Key Laboratory of Pediatric Metabolism and Inflammatory Diseases, Chongqing 400014, China

**Keywords:** neuroblastoma, prognosis, lactate dehydrogenase-to-albumin ratio, lactate dehydrogenase, albumin

## Abstract

Purpose: This study aimed to investigate the association between lactate dehydrogenase-to-albumin ratio (LAR) and the clinical characteristics and overall survival (OS) of patients with neuroblastoma (NB). Methods: We conducted a retrospective data analysis of 443 patients diagnosed with neuroblastoma. The optimal cut-off value for the LAR was determined using receiver operating characteristic (ROC) curves. We utilized Kaplan–Meier curves and Cox regression analysis to evaluate the relationship between LAR and OS. Independent factors identified through multivariate analysis were employed to construct a nomogram. The performance of the nomogram model was assessed using calibration curves, ROC curves, concordance index (C-index), and decision curve analysis (DCA). Results: The 2-year time-dependent ROC curve indicated that the optimal cut-off value for the LAR was 21.814. Kaplan–Meier survival curve analysis revealed that the prognosis for the high LAR group was significantly worse than that for the low LAR group. Results from multivariate Cox analysis identified INSS stage, bone metastasis, *MYCN*, and LAR as independent prognostic factors for OS. A nomogram for predicting the prognosis of NB was established based on multivariate Cox regression analysis. Internal validation through the Bootstrap method revealed that the nomogram’s C-index was 0.727. Both the calibration curve and ROC curve suggested that the model possessed significant predictive potential. DCA further demonstrated that the nomogram model exhibited substantial clinical applicability. Conclusions: LAR served as an aussichtsreich prognostic indicator for neuroblastoma, and the nomogram model based on LAR can predict the OS of patients with this condition.

## 1. Introduction

Neuroblastoma is the predominant extracranial solid tumor in pediatric patients, originating from the adrenal medulla or sympathetic nervous system. This malignancy exhibits significant heterogeneity, characterized by distinct biological and clinical features. While low-to-intermediate-risk neuroblastomas can often be treated effectively through surgical resection, high-risk neuroblastoma is typically associated with a poor prognosis. Despite advancements in multimodal therapeutic strategies, the 5-year survival rate for high-risk patients has remained below 50% in recent years [1,2,3]. Therefore, clinicians must identify accessible and relatively accurate prognostic indicators to enhance risk assessment.

Aerobic glycolysis, commonly termed the Warburg effect, is a metabolic hallmark of cancer cells that facilitates their proliferation and metabolic expansion. LDH, a key enzyme in glycolysis, has been shown to have elevated serum levels that are frequently correlated with unfavorable prognoses in various malignancies [4,5,6]. Accumulating evidence has demonstrated that lactate dehydrogenase (LDH) facilitates the identification of high-risk neuroblastoma patients with unfavorable prognoses and confers independent prognostic significance [7]. However, LDH, a well-investigated enzyme released from injured and necrotic tissues, exhibits insufficient diagnostic specificity in differentiating between normal and tumor tissues [7,8,9]. As a frequently used indicator for evaluating nutritional status among cancer patients, serum albumin (ALB) has gained increasing attention. Recent investigations have emphasized that malnutrition acts as a key predictive factor for survival in this patient population, with ALB levels exerting substantial influence on tumor prognosis [10,11].

The LDH/ALB ratio (LAR) was emerging as a novel tumor biomarker, with prior research predominantly centered on adult malignant tumors. For instance, LAR served as an independent predictor of complications in colon cancer, as well as overall survival and disease-free survival. In the context of bladder cancer, LAR was recognized as a reliable and independent biomarker for postoperative survival and prognosis [12,13,14]. Furthermore, LAR also correlated with the prognosis of patients suffering from lower respiratory infections and sepsis [15,16]. However, as a pediatric embryonic tumor with distinct biological and clinical characteristics, neuroblastoma cannot have its prognostic marker exploration simply extrapolated from adult models. The present study aims to specifically evaluate the prognostic value of LAR in this childhood-specific malignancy, thereby addressing the existing gap in current knowledge.

Currently, there is no existing report on the relationship between LAR and the prognosis of pediatric neuroblastoma patients. Consequently, this study retrospectively analyzed the impact of LAR on the prognosis of 443 neuroblastoma patients to evaluate the prognostic predictive value of LAR. Additionally, we assessed the effectiveness of the survival prediction nomogram incorporating LAR, which can assist in the effective stratification of risks and enhance personalized therapeutic regimens, thereby providing valuable guidance for clinical management.

## 2. Materials and Methods

### 2.1. Patients

A total of 443 pediatric patients diagnosed with neuroblastoma via pathological confirmation or bone marrow biopsy/puncture were enrolled from the Children’s Hospital of Chongqing Medical University between June 2012 and March 2022. Exclusion criteria encompassed patients with incomplete clinical records, including missing data on symptoms, signs, or ancillary examinations at initial diagnosis. Tumor staging was established according to the International Neuroblastoma Staging System (INSS), while risk stratification was performed based on the Children’s Oncology Group (COG) classification system. Long-term prognosis was assessed by overall survival (OS), with follow-up data collected until December 2023. The study protocol was approved by the Ethics Committee of the Children’s Hospital of Chongqing Medical University (Approval No: 2019-235).

### 2.2. Data Collection

This retrospective study documented the clinicopathological profiles of enrolled patients, encompassing demographic data (age and sex), diagnostic details, INSS staging, primary tumor location, sites of metastasis (with specific reference to osseous and bone marrow involvement), *MYCN* (amplified vs. non-amplified), and histopathological subtype. Furthermore, pre-treatment serum levels of albumin (ALB) and lactate dehydrogenase (LDH), as well as 24 h urinary vanillylmandelic acid (VMA) excretion, were recorded. The lactate dehydrogenase-to-albumin ratio (LAR) was derived from the formula: LAR = LDH (U/L)/ALB (g/L).

### 2.3. Treatment

Treatment strategies were determined according to the COG risk stratification criteria. Patients categorized as low- to intermediate-risk generally underwent surgical resection as the main intervention, supplemented by chemotherapy. Those with a solitary and localized tumor received one-stage complete resection. For patients deemed ineligible for immediate surgery, 2–4 cycles of neoadjuvant chemotherapy were administered preoperatively to reduce tumor burden. In the low-risk group, adjuvant chemotherapy consisted of 2–4 cycles, whereas the intermediate-risk group received prolonged chemotherapy for 4–6 cycles after surgery. High-risk patients were managed with multimodal therapy, which included surgery, chemotherapy, adjuvant radiotherapy, Isotretinoin, and traditional Chinese medicine. In this subgroup, 3–4 cycles of preoperative chemotherapy were followed by 4–6 cycles of postoperative chemotherapy. The chemotherapeutic regimens commonly employed in our department comprised agents such as cyclophosphamide, vincristine, cisplatin, etoposide, and doxorubicin.

### 2.4. LDH and ALB Level Detection

The serum LDH level was determined by the lactate substrate rate method using a CIBACorning-560 automatic biochemical analyzer (Corning Inc., Corning, NY, USA), and the ALB was measured by the bromocresol green method.

### 2.5. Optimal Cut-Off Value and Groups

LAR was derived by dividing serum LDH (U/L) by serum albumin (g/L). The optimal threshold for LAR was determined via a 2-year receiver operating characteristic (ROC) curve analysis. The Youden index, calculated as sensitivity + specificity − 1, was maximized at a cutoff value of 21.814. Death was used as the event and time-dependent ROC analysis accounts for using the time ROC package and ggplot2 at t = 2 years. Censored data are processed using the Kaplan–Meier method. Accordingly, patients were stratified into a low-LAR group (LAR < 21.814) and a high-LAR group (LAR ≥ 21.814).

### 2.6. Statistical Analysis

Categorical variables were compared using the Chi-square test. Survival outcomes were evaluated with Kaplan–Meier curves, and differences between groups were examined using the log-rank test. To identify prognostic factors for OS, univariate Cox regression analyses were performed. Variables yielding *p* < 0.1 in univariate analysis were subsequently entered into a multivariate Cox regression model for further adjustment. Based on the final multivariate results, a nomogram was constructed to predict OS. The predictive performance of the nomogram was validated through calibration curves, Harrell’s concordance index (C-index), and receiver operating characteristic (ROC) curves. Decision curve analysis (DCA) was further applied to assess its clinical net benefit. All analyses were conducted using SPSS 27.0 (IBM Corp., Armonk, NY, USA) and R 4.2.1 (http://www.R-project.org, accessed on 4 June 2024), with statistical significance set at a two-tailed *p* < 0.05.

## 3. Results

### 3.1. Clinical Characteristics of Patients

This study retrospectively collected the clinical characteristics from 443 patients diagnosed with neuroblastoma. The clinical characteristics of the patients are shown in Table 1. The median age at initial diagnosis was 30 months (range: 0.1–156 months), and the median follow-up time was 20 months (range: 1–115 months). According to the LAR cut-off value of 21.814 (Table S1), 332 patients were categorized into the low LAR group, while 111 patients were assigned to the high LAR group.

No significant differences were observed in gender (*p* = 0.129), age (*p* = 0.139), serum ALB level (*p* = 0.870), or 24 h urinary VMA (*p* = 0.337). In contrast, significant variations (*p* < 0.001) existed in primary tumor site, INSS stage, COG risk classification, Shimada type, *MYCN*, bone metastasis, bone marrow metastasis, and serum LDH level, as shown in Table 1.

### 3.2. LAR in the Prediction of Prognosis for Neuroblastoma

**Figure 1:**
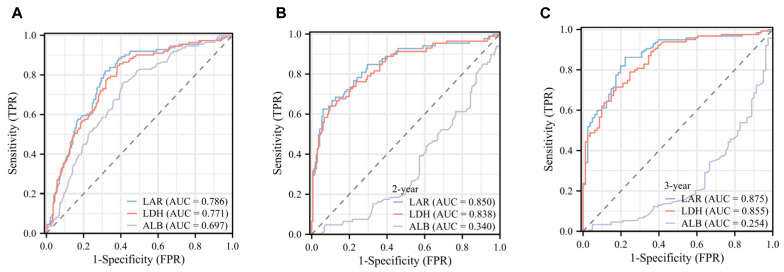
(**A**) Receiver operating characteristic (ROC) curves for LAR, LDH, and ALB. (**B**,**C**) Time-dependent ROC curves for 2 years and 3 years, respectively. The dashed diagonal line in each ROC plot serves as the random guessing reference baseline, corresponding to a model with no diagnostic or predictive value (AUC = 0.5). Markers whose curves lie above this line demonstrate better-than-random discriminatory ability, with greater deviation from the dashed line indicating stronger performance.

As depicted in Figure S3A, restricted cubic spline (RCS) modeling revealed a non-linear relationship between LAR (a continuous variable) and mortality risk, with LAR emerging as an important prognostic indicator. ROC curve analysis demonstrated that LAR outperformed LDH and ALB in predicting overall survival (OS) when each marker was evaluated individually (Figure 1A). The corresponding area under the curve (AUC) values were 0.786 (95% CI: 0.738–0.833) for LAR, 0.771 (95% CI: 0.721–0.820) for LDH, and 0.697 (95% CI: 0.643–0.751) for ALB. Youden’s indices for LAR, LDH, and ALB were 0.57277, 0.53982, and −0.30645, respectively (Table S1).

Time-dependent ROC analysis further assessed the predictive performance of LAR, LDH, and ALB for 2-year and 3-year OS. Consistent with the static ROC results, LAR showed better prognostic accuracy across all time points, with AUC values of 0.850 and 0.875, respectively. In comparison, LDH yielded AUCs of 0.838 and 0.855, while ALB exhibited notably lower AUCs of 0.340 and 0.254 (Figure 1B,C).

### 3.3. Kaplan–Meier Survival Curves for OS

Kaplan–Meier analysis indicated a significantly poorer prognosis in the high LAR group compared with the low LAR group (Figure S1A). Similarly, patients with *MYCN* amplification showed worse survival outcomes than those without amplification (Figure S1B). The INSS stage 4 also exhibited an inferior prognosis relative to the non-stage 4 patients (Figure S1C). Furthermore, the presence of bone metastasis was associated with poorer survival compared with its absence (Figure S1D).

### 3.4. The Prognostic Value of LAR Was Independent of Age in Neuroblastoma

Spearman’s correlation analysis between age and LDH levels in our cohort revealed no significant association (R = 0.004, *p* = 0.928) (Figure S2A), suggesting that age may not be a major driver of LAR within the age range examined. Subgroup analysis, stratified by age >18 months versus ≤18 months, demonstrated that LAR retained its ability to effectively distinguish prognostic outcomes across both age subgroups (Figure S2B,C).

### 3.5. Univariate and Multivariate Cox Regression Analysis for OS

Cox regression analysis was performed to identify independent predictors of OS. In the univariate analysis, age, primary site, INSS stage, Shimada type, bone marrow metastasis, bone metastasis, *MYCN*, serum LDH, ALB, LAR, and 24 h urinary VMA were all significantly associated with OS (all *p* < 0.01) (Table 2). In the subsequent multivariate model, which included these variables, INSS stage, bone metastasis, *MYCN*, and LAR emerged as independent prognostic factors for OS (all *p* < 0.05) (Table 2). The original data consisted of 443 entries. There were 78 samples with missing variable information in the Cox regression analysis. The final number of samples included: 365. As a default approach, specimens with variable missing values were consistently excluded before conducting statistical analysis, without implementing any imputation procedures. Variance inflation factors (VIF) for LAR, LDH, and ALB were all below the conventional threshold of 4, indicating no substantive multicollinearity, as shown in Table S4.

### 3.6. Construction and Evaluation of the Nomogram Model

**Figure 2:**
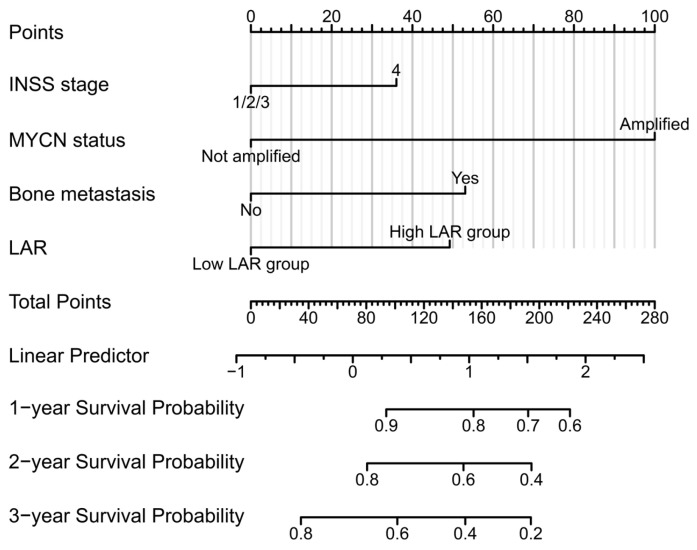
Nomogram model for OS. The line segment representing each variable was marked with a scale that indicated the range of possible values for that variable. A vertical line was drawn upward to obtain the corresponding score for each variable. By summing all individual variable scores, we can derive the total score, from which a vertical line was subsequently drawn downward to determine the probability of OS at 1, 2, or 3 years.

Based on the multivariate Cox regression results, four variables—INSS stage, bone metastasis, *MYCN*, and LAR—were selected to construct a nomogram for predicting 1-, 2-, and 3-year OS (Figure 2). There were 72 samples with missing variable information in the nomogram model. The final number of samples included: 371. Each variable was assigned a score, and the total score was used to stratify patients into distinct prognostic groups. Assessment of proportional hazards via Schoenfeld residuals revealed no significant violations across all covariates (all *p* > 0.05), as shown in Table S6.

**Figure 3:**
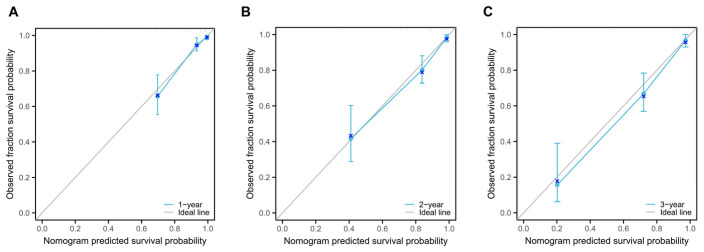
Calibration curves for the nomograms model of OS at 1 year (**A**), 2 years (**B**), and 3 years (**C**).

**Figure 4:**
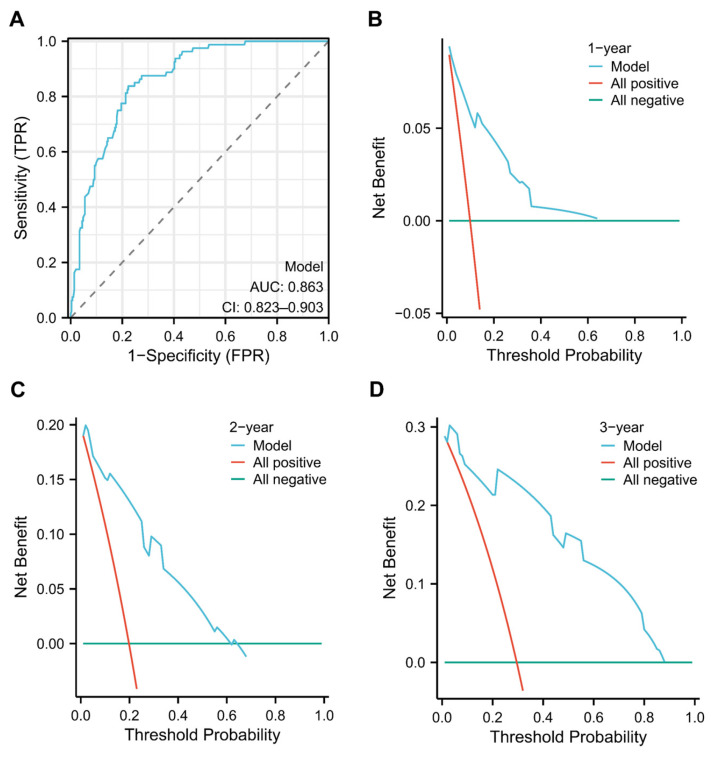
(**A**) ROC curve of the nomogram model for OS prediction. The blue line represents the ROC curve of the nomogram model (AUC = 0.863, 95% CI: 0.823–0.903), demonstrating its discriminatory performance for OS. The dashed diagonal line serves as the random guessing baseline (AUC = 0.5), providing a reference for models with no predictive value. (**B**–**D**) Decision curve analyses (DCA) for predicting OS at 1 year, 2 years, and 3 years, respectively. In these DCA plots, the blue line denotes the net benefit of the nomogram model across varying threshold probabilities, reflecting its clinical utility. The red line represents the “All positive” strategy (classifying all patients as positive), and the turquoise line represents the “All negative” strategy (classifying all patients as negative), both serving as reference benchmarks for evaluating the model’s net clinical value.

To validate the nomogram’s performance (full model development), bootstrap resampling (1000 iterations) was performed for internal validation. The model’s concordance index (C-index) was 0.727 (95% CI, 0.696–0.757). Calibration curves for 1-, 2-, and 3-year OS demonstrated close alignment between predicted and observed outcomes, indicating good model calibration (Figure 3A–C). Receiver operating characteristic (ROC) curve analysis further evaluated the nomogram’s discriminative ability. The model showed strong predictive performance, with an area under the curve (AUC) of 0.863 (95% CI, 0.823–0.903) (Figure 4A). Decision curve analysis (DCA) was used to assess clinical utility. For 1-year OS, the nomogram provided a net benefit across a threshold probability range of 10% to 60% (Figure 4B). For 2- and 3-year OS, the model significantly improved net benefit within a threshold range of 10% to 80% (Figure 4C,D). Collectively, these findings indicate that the nomogram-based risk prediction model exhibits favorable predictive accuracy and clinical applicability.

## 4. Discussion

In this study, we believe that LAR may better reflect the dynamic balance between tumor burden (as indicated by LDH) and the patient’s overall nutritional/inflammatory status (as indicated by ALB). This “high metabolic burden and low nutritional reserve” state of neuroblastoma may be more capable of capturing the aggressiveness of the disease and the vulnerability of the patient than looking at either indicator alone. Additionally, it is the simplicity of clinical application: a single, comprehensive ratio-based indicator is easier to understand and implement in clinical practice than two separate markers that require concurrent interpretation, with one portending poorer outcomes at higher values (LDH) and the other at lower values (ALB).

Survival analyses revealed that patients in the high LAR group had significantly poorer OS outcomes than those in the low LAR group. Multivariable Cox regression further confirmed that LAR was an independent predictor of OS. We therefore developed a LAR-integrated nomogram and validated its predictive accuracy and clinical utility. To our knowledge, this study is the first to investigate the prognostic value of LAR in neuroblastoma.

Lactate dehydrogenase (LDH) plays a pivotal role in tumor cell metabolism, specifically functioning as the key enzyme that mediates the conversion of pyruvate to lactate in the glycolytic pathway. Conversely, albumin (ALB) acts as a reliable biomarker for evaluating the systemic nutritional status of cancer patients, a factor frequently associated with clinical outcomes. Accumulating evidence suggests that elevated LDH activity, in conjunction with reduced ALB levels, is correlated with diminished survival rates across a spectrum of malignancies [17,18,19,20,21,22,23]. The crosstalk between LDH and ALB mirrors an individual’s inflammatory burden and nutritional status. Notably, the LAR exhibited superior performance in survival prediction compared to either LDH or ALB as standalone markers, a conclusion corroborated by our experimental data. Furthermore, accumulating evidence has demonstrated that LAR outperformed the TNM staging system with respect to OS and disease-free survival (DFS) in colorectal cancer (CRC) patients. Clinical predictive models integrated with LAR hold the potential to augment the clinical benefits related to OS and DFS among CRC populations [24]. Among patients diagnosed with diffuse large B-cell lymphoma (DLBCL), increased LAR concentrations were found to be substantially associated with diminished OS and progression-free survival (PFS) [25]. Additionally, high levels of the LAR prior to surgery have been identified as an independent prognostic factor in breast cancer patients [26].

Over the past decade, there has been a rising emphasis on risk stratification and outcome evaluation in patients with neuroblastoma, as therapeutic regimens are tailored to match individual risk characteristics. Factors closely linked to neuroblastoma prognosis encompass patient age, degree of tumor differentiation, MYCN status, and particular chromosomal segmental aberrations [27,28,29]. In this research, multivariate Cox regression analysis demonstrated that INSS stage, presence of bone metastasis, *MYCN* amplification, and LAR served as independent predictors of OS. After confirming these factors’ independent prognostic value, a nomogram model was constructed for OS prediction, and its accuracy as well as clinical applicability were validated through relevant analyses. The value of this study lies in identifying that, beyond the classic COG risk stratification framework, LAR can provide independent prognostic information with incremental value. As an easily accessible blood marker, it serves as a useful supplement to existing complex stratification systems, aiding clinicians in conducting more refined risk assessment and management during the initial stage and course of treatment.

This study defined the high LAR group as LAR ≥ 21.814 and the low LAR group as LAR < 21.814, utilizing the 2-year time-dependent ROC curve. It is important to emphasize that the determination of LAR cutoff values primarily relies on ROC and restricted cubic spline (RCS) analyses [12,16,24,25,26]. However, reported cutoff values vary across studies, influenced by factors such as tumor type, sample size, and measurement techniques. Therefore, establishing definitive cutoff values for neuroblastoma will require larger, multicenter clinical studies.

This study has several limitations. First, its retrospective, single-center design may introduce selection bias, and the control of certain unmeasured potential confounding factors—such as specific comorbidities or detailed treatment information—was limited. Second, the median follow-up duration of 20 months is relatively short, which restricts the ability of the model to evaluate long-term survival outcomes, including the 5-year overall survival rate. Third, although the management of high-risk patients in this study was generally consistent with established clinical guidelines, the heterogeneity of treatment approaches and their potential impact on prognosis were not fully explored in the present analysis. Fourth, despite the model demonstrating good discrimination and internal calibration, further validation in multicenter, prospective cohorts or using external data is required to confirm its generalizability. Finally, the included biological markers may be influenced by non-tumor factors such as inflammation and nutritional status, and their interpretation in the context of pediatric neuroblastoma should therefore be made with caution. Future studies should aim to extend follow-up time, standardize data collection, and conduct external validation to further clarify the clinical applicability and incremental value of this predictive tool.

## 5. Conclusions

LAR holds promise as a clinical research and hypothesis-generating tool. We have initially developed and internally validated a prognostic nomogram, which exhibits promising predictive potential. However, its path to clinical application mandates rigorous prospective validation in multicenter settings.

## Figures and Tables

**Table 1:** Baseline characteristics of patients with neuroblastoma.

Characteristics	Low LAR Group	High LAR Group	*p* Value
***n***	332	111	
**Gender, *n* (%)**			0.129
Female	150 (45.2%)	41 (36.9%)	
Male	182 (54.8%)	70 (63.1%)	
**Age (months), *n* (%)**			0.139
≤18	114 (34.3%)	31 (27.9%)	
>18	218 (65.7%)	80 (72.1%)	
**Primary site, *n* (%)**			<0.001
Adrenal glands/Retroperitoneum	218 (65.7%)	105 (94.6%)	
Mediastinum	94 (28.3%)	6 (5.4%)	
Other sites	20 (6%)	0 (0%)	
**INSS stage, *n* (%)**			<0.001
1/2/3	189 (56.9%)	11 (9.9%)	
4	143 (43.1%)	100 (90.1%)	
**COG risk classification, *n* (%)**			<0.001
Non-high risk	167 (50.3%)	2 (1.8%)	
High-risk	165 (49.7%)	109 (98.2%)	
**Shimada Type, *n* (%)**			<0.001
FH	159 (47.9%)	12 (10.8%)	
uFH	149 (44.9%)	79 (71.2%)	
Unknown	24 (7.2%)	20 (18%)	
***MYCN*, *n* (%)**			<0.001
Not amplified	273 (82.2%)	37 (33.3%)	
Amplified	21 (6.3%)	60 (54.1%)	
Unknown	38 (11.5%)	14 (12.6%)	
**Bone marrow metastasis, *n* (%)**			<0.001
No	241 (72.6%)	41 (36.9%)	
Yes	91 (27.4%)	70 (63.1%)	
**Bone metastasis, *n* (%)**			<0.001
No	234 (70.5%)	41 (36.9%)	
Yes	78 (23.5%)	55 (49.5%)	
Unknown	20 (6%)	15 (13.5%)	
**Serum LDH level (U/L), *n* (%)**			<0.001
≥1500	25 (7.5%)	43 (38.7%)	
<1500	307 (92.5%)	68 (61.3%)	
**Serum ALB level (g/L), *n* (%)**			0.870
≥35	295 (88.9%)	98 (88.3%)	
<35	37 (11.1%)	13 (11.7%)	
**Urine VMA (mg/24 h), *n* (%)**			0.337
>13.6	155 (46.7%)	46 (41.4%)	
≤13.6	177 (53.3%)	65 (58.6%)	

Abbreviations: LAR, lactate dehydrogenase-to-albumin ratio; INSS, International Neuroblastoma Staging System; COG, Children’s Oncology Group; FH, favorable histology; uFH, unfavorable histology; LDH, lactate dehydrogenase; VMA, vanillylmandelic acid; ALB, albumin.

**Table 2:** Univariate and multivariate Cox regression analysis for OS.

Characteristics	Total (N)	Univariate Analysis		Multivariate Analysis	
		Hazard Ratio (95% CI)	*p* Value	Hazard Ratio (95% CI)	*p* Value
Gender	443				
Female	191	Reference			
Male	252	1.363 (0.928–2.003)	0.115		
Age (months)	443				
>18	298	Reference		Reference	
≤18	145	0.286 (0.172–0.476)	<0.001	0.562 (0.253–1.249)	0.157
Primary site	443				
Adrenalglands/ Retroperitoneum	323	Reference		Reference	
Mediastinum	100	0.314 (0.168–0.587)	<0.001	0.364 (0.141–0.942)	0.037
Other sites	20	0.394 (0.125–1.245)	0.113	0.828 (0.168–4.067)	0.816
INSS stage	443				
1/2/3	200	Reference		Reference	
4	243	13.804 (7.172–26.572)	<0.001	8.926 (2.811–28.350)	<0.001
Shimada Type	399				
FH	171	Reference		Reference	
uFH	228	3.464 (2.104–5.703)	<0.001	0.750 (0.369–1.525)	0.427
*MYCN*	391				
Not amplified	310	Reference		Reference	
Amplified	81	5.366 (3.499–8.230)	<0.001	2.954 (1.693–5.154)	<0.001
Bone marrow metastasis	443				
Yes	161	Reference		Reference	
No	282	0.219 (0.147–0.326)	<0.001	1.166 (0.591–2.299)	0.657
Bone metastasis	408				
No	275	Reference		Reference	
Yes	133	8.431 (5.292–13.431)	<0.001	3.581 (1.753–7.315)	<0.001
Serum LDH level (U/L)	443				
<1500	375	Reference		Reference	
≥1500	68	6.347 (4.161–9.681)	<0.001	1.676 (0.821–3.420)	0.156
Serum ALB level (g/L)	443				
≥35	393	Reference		Reference	
<35	50	2.246 (1.383–3.649)	0.001	1.560 (0.831–2.930)	0.166
LAR	443				
Low LAR group	332	Reference		Reference	
High LAR group	111	2.542 (1.649–3.918)	<0.001	2.728 (1.445–5.151)	0.002
Urine VMA (mg/24 h)	443				
≤13.6	242	Reference		Reference	
>13.6	201	1.689 (1.159–2.463)	0.006	0.708 (0.353–1.418)	0.330

Abbreviations: LAR, lactate dehydrogenase-to-albumin ratio; INSS, International Neuroblastoma Staging System; FH, favorable histology; uFH, unfavorable histology; LDH, lactate dehydrogenase; VMA, vanillylmandelic acid; ALB, albumin.

## Data Availability

All data generated or analyzed during this study are included in the article and its Supplementary Files, and are available from the corresponding author on reasonable request.

## Supplementary Materials

The following supporting information can be downloaded at https://www.mdpi.com/article/10.3390/children13020220/s1. Table S1: Results from 2-year time-dependent receiver operating characteristic analysis. Table S2: AUC result table in Figure 1A. Table S3: Statistical description of time-dependent ROC. Table S4: Variance Inflation Factor (VIF) in Cox regression analysis. Table S5: Supplementary information on Cox regression analysis in Nomogram. Table S6: Proportional hazards (PH) of Cox regression analysis in Nomogram. Figure S1: Kaplan–Meier survival curves for overall survival (OS) in (A) High LAR and low LAR groups. (B) MYCN amplified and non-amplified groups. (C) INSS stage 4 and INSS stage 1/2/3 group. (D) bone metastasis and non-bone metastasis group. Figure S2: (A) Correlation analysis of LAR and age. (B) Kaplan–Meier survival curves compare OS between high- and low-LAR groups in patients aged >18 months. (C) Kaplan–Meier survival curves compare OS between high- and low-LAR groups in patients aged ≤18 months. Figure S3: (A) Restricted Cubic Spline (RCS) plot of LAR (with hazard ratios).
