# Supplementary material for: Utilizing the Lactate Dehydrogenase-to-Albumin Ratio for Survival Prediction in Patients with Neuroblastoma
Source: Children (Basel). 2026 Feb 4;13(2):220. doi: 10.3390/children13020220 (PMC12940032; doi:10.3390/children13020220)
Supplement: Supplementary file 1 [file children-13-00220-s001.zip › children-4104823-supplementary.pdf]

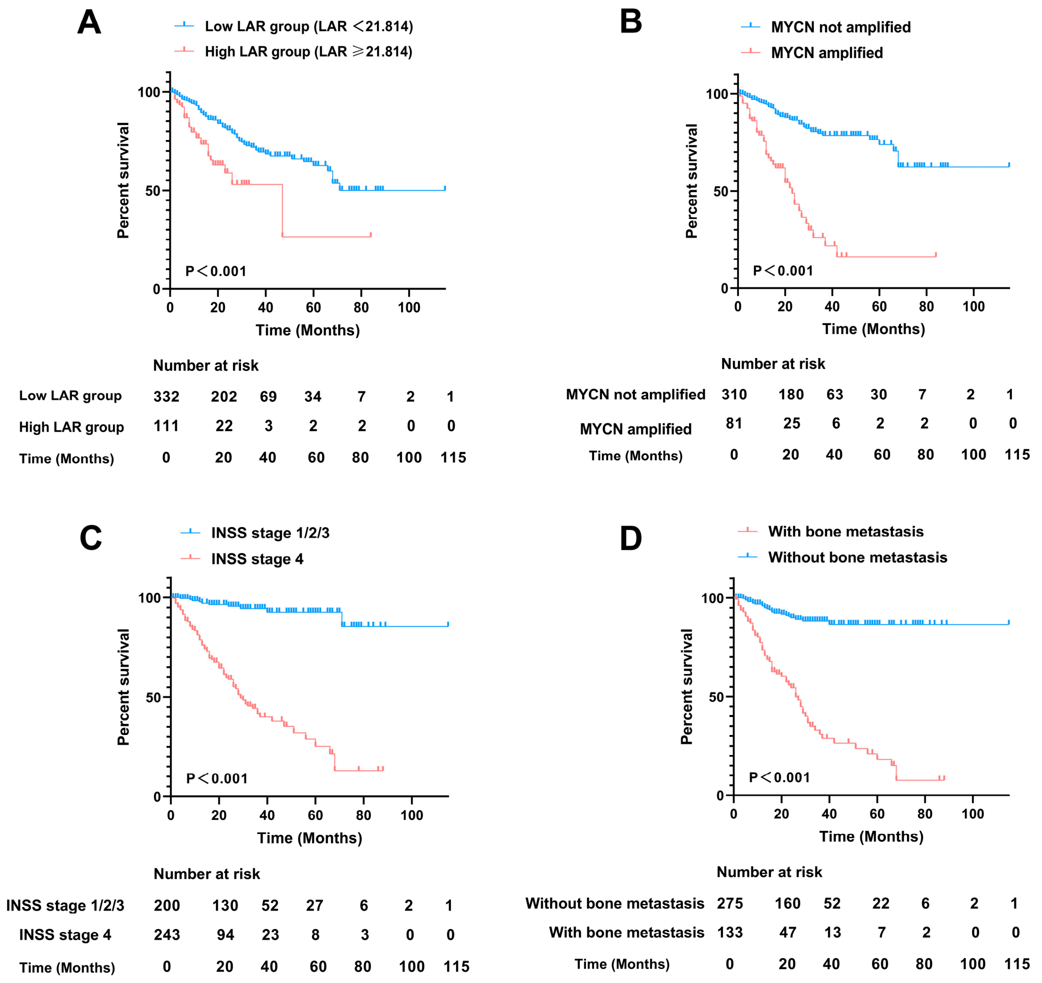

Figure S1. Kaplan–Meier survival curves for overall survival (OS) in (A) High LAR and low LAR groups. (B) MYCN amplified and non-amplified groups. (C) INSS stage 4 and INSS stage 1/2/3 group. (D) bone metastasis and non-bone metastasis group.

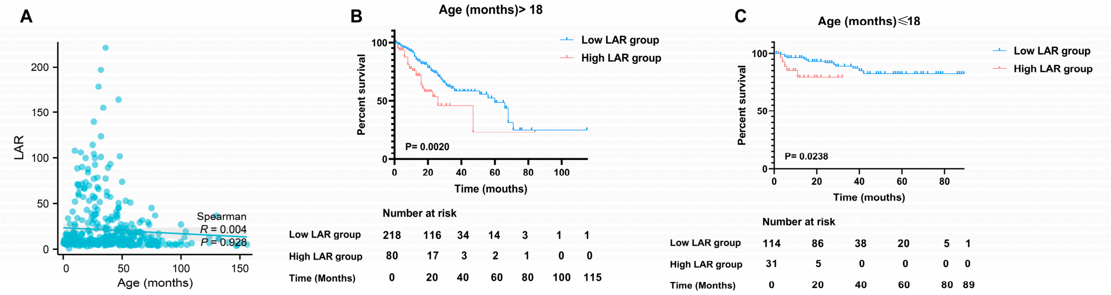

Figure S2. (A) Correlation analysis of LAR and age. (B) Kaplan-Meier survival curves compare OS between high- and low-LAR groups in patients aged >18 months. (C) Kaplan-

Meier survival curves compare OS between high- and low-LAR groups in patients aged ≤18 months.

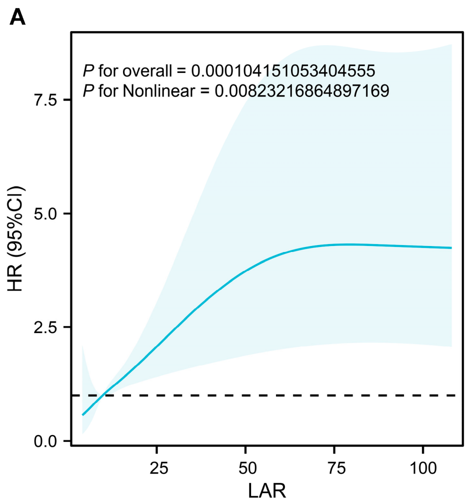

Figure S3. (A) Restricted Cubic Spline (RCS) plot of LAR (with hazard ratios).

Table S1. Results from 2-years time-dependent receiver operating characteristic analysis.

Variables	Cut-off value	Sensitivity	Specificity	Positive predictive value	Negative Predictive value	Youden index
LAR	21.814	0.63301	0.93976	0.76165	0.89385	0.57277
LDH	730.4	0.63621	0.90361	0.66748	0.89092	0.53982
ALB	44.1	0.18753	0.50602	0.1035	0.67192	-0.30645

Abbreviations: LAR, lactate dehydrogenase-to-albumin ratio; LDH, lactate dehydrogenase; ALB, albumin.

Table S2. AUC result table in Figure 1A

Predictor variable	Predicted outcome	AUC	CI
LAR	Positive	0.786	0.738 – 0.833
LDH	Positive	0.771	0.721 – 0.820
ALB	Negative	0.697	0.643 – 0.751

Table S3. Statistical description of time-dependent ROC

Years	Number of deaths	Survival number	Censored number
2	82	166	195
3	100	81	262
5	107	29	307

Table S4. Variance Inflation Factor (VIF) in Cox regression analysis

Variable	Type	Count by Category	VIF
Serum LDH level (U/L)	Categorical variable	2	1.4663
Serum ALB level (g/L)	Categorical variable	2	1.1609
LAR	Categorical variable	2	1.1819

Description: Variance inflation factors for LAR, LDH, and ALB were all below the conventional threshold of 4, indicating no substantive multicollinearity.

Table S5. Supplementary information on Cox regression analysis in Nomogram

Variable	Missing numbers	number of events	Coefficient β
LAR	0	443	0.61009
INSS stage	0	443	0.44645
MYCN status	52	391	1.2392
Bone metastasis	35	308	0.65773

Description: The original data consisted of 443 entries. There were 72 samples with missing variable information in nomogram model. The final number of samples included: 371. As a default approach, specimens with variable missing values were consistently excluded before conducting statistical analysis, without implementing any imputation procedures.

Table S6. Proportional hazards (PH) of Cox regression analysis in Nomogram

Variable	Chi-Square	Degrees of Freedom (df)	P value
INSS stage	0.035985	1	0.8495
MYCN	0.0040102	1	0.9495
Bone metastasis	2.066	1	0.1506
LAR	1.1281	1	0.2882
GLOBAL	3.6928	4	0.4492
